# The Role of Preradiosurgical Embolization in the Management of Grades III, IV, and V Arteriovenous Malformations

**DOI:** 10.3389/fsurg.2016.00037

**Published:** 2016-06-28

**Authors:** Evandro C. Sousa, Manoel J. Teixeira, Ronnie L. Piske, Lavoisier S. Albuquerque, Sebastião Côrrea, Salomão Benabou, Leonardo C. Welling, Leonardo Moura de Sousa, Eberval Gadelha Figueiredo

**Affiliations:** ^1^Division of Neurological Surgery, University of São Paulo, São Paulo, Brazil; ^2^Hospital Beneficência Portuguesa, São Paulo, Brazil

**Keywords:** arteriovenous malformation, embolization, radiosurgery, staged embolization, surgery

## Abstract

**Objective:**

To evaluate the role of preradiosurgical embolization on obliteration rate, reduction of size, irradiation dose, and neurological outcome, in 90 patients presenting large arteriovenous malformations (AVMs).

**Methods:**

Between October 1993 and October 2006, 90 radiosurgical procedures were performed to treat brain AVMs Spetzler–Martin (SM) grades III, IV, and V at the Department of Radiosurgery and Radiology of the Real e Benemérita Associação Portuguesa de Beneficência de São Paulo, São Paulo, Brazil. Fifty-nine patients had embolization before radiosurgery and complete clinical and radiologic follow-up for at least 3 years. Inclusion criteria were as follow: SM grades III, IV, and V AVMs, no previous treatment, and clinical and radiological (angiogram and MRI) follow-up for at least 3 years. Obliteration rate, reduction of size, irradiation dose, and neurological outcome were compared in these two cohorts of patients. Mann–Whitney test, “Student’s *t*-test,” and χ^2^ tests were used for statistical analysis, as appropriate. The level of significance was determined at *p* < 0.05.

**Results:**

The mean size of the AVMs that underwent embolization was significantly greater when compared with non-embolized group (*p* < 0.05). Embolization significantly reduced the AVM diameter. Irradiation dose was significantly smaller in the embolized group (*p* < 0.05). No significant differences in final clinical outcomes, postprocedural radiological findings, rate of occlusion, and need for additional procedures were observed between the two groups (*p* < 0.05).

**Conclusion:**

Preradiosurgical embolization of large AVMs does not result in impaired obliteration rate compared with cases treated with radiosurgery alone. It did not add further morbidity and presented benefits of reducing size of the AVMs. Preradiosurgical embolization may facilitate the coverage of the AVM with the effective irradiation dose. Combined management may be effective for selected large lesions considered unsuitable for radiosurgery and otherwise untreatable.

## Introduction

Intracranial arteriovenous malformations (AVMs) constitute a formidable medical challenge. When amenable, surgical treatment is the best option because it affords immediate cure and prevents hemorrhage. However, surgery is not always feasible and frequently additional therapeutic modalities, such as embolization and radiotherapy, are required. Radiosurgery may be employed as an alternative to microsurgery in small lesions or as a part of a combined treatment of Spetzler–Martin (SM) grades III, IV, and V AVMs. However, it does not always obliterate the nidus completely (1–21). The occlusion rate varies proportionally with the inverse of the AVM’s diameter. Therefore, large AVMs are not suitable for radiosurgical treatment unless their size is reduced.

In thesis, initial volume reduction with endovascular embolization is useful in large AVMs and may facilitate complete obliteration with therapeutically effective dose. However, the role of preradiosurgical embolization is still to be definitively established. Most of the reports actually indicate that it reduces the obliteration rate and increases morbidity (2, 6, 14, 22), and many authors indeed do not recommend preradiosurgical embolization. Therefore, a large number of patients are left untreated.

In this paper, we retrospectively investigated 90 patients presenting III, IV, and V SM grade AVMs, treated with or without preradiosurgical embolization between 1993 and 2006, aiming to evaluate the role of this procedure on obliteration rate, reduction of size, need for additional procedures, irradiation dose, and neurological outcome.

## Materials and Methods

Between October 1993 and October 2006, 90 radiosurgical procedures were performed to treat brain AVMs SM grades III, IV, and V, at the Department of Radiosurgery and Radiology of the Real e Benemérita Associação Portuguesa de Beneficência de São Paulo, São Paulo, Brazil. A multidisciplinary team consisting of vascular neurosurgeons, endovascular therapists, and radiosurgeons initially was involved in this study. Of these 90 patients, 59 had embolization before radiosurgery and complete clinical and radiologic follow-up for at least 3 years. AVMs were classified according to the SM grading system by neurosurgeons, interventional radiologists, and radiosurgeons.

Forty-six (51.1%) patients were female and 44 (48.9%) were male. Mean age was 30.6 years. Asymptomatic AVMs are composed of 7.8% (seven cases). Inclusion criteria were SM grades III, IV, and V AVMs, AVMs with no previous treatment, and patients with clinical and radiological (angiogram and MRI) follow-up for at least 3 years. From the original cohort of 105 patients, 15 did not meet the inclusion criteria.

Clinical presentation is detailed in Table 1. Forty-one (45.6%) patients presented with hemorrhage (Table 2). Fifty-two patients (57.8%) presented angioarchitectural abnormalities, including intranidal aneurysms, arterial or venous stenosis, and venous ectasia. Occlusion rate, irradiation doses, clinical morbidity, and radiological complications were evaluated and compared between the two groups.

**Table 1:** **Clinical presentation of the patients (*n* = 90)**.

Clinical presentation	*n*	%
Paresis	41	45.6
Headache	35	38.9
Seizures	33	36.7
Incidental	07	7.8
Cognitive dysfunction	03	1.1

**Table 2:** **Ocurrence of hemorraghe at clinical presentation (*n* = 90)**.

Hemorraghe	*n*	%
Absente	49	54.4
Intraparenchymal	21	23.3
Intraparenchymal and intraventricular	15	16.7
Subarachnoid hemorraghe	02	2.2
Subarachnoid hemorraghe and Intraparenchymal	02	2.2
Intraparenchymal, intraventricular and subarachnoid hemorraghe	01	1.1

### Endovascular Procedure

The goals of the procedure were to decrease the size of lesion and target lesions that would increase the risks for hemorrhage, such as aneurysms. All procedures were carried out under general anesthesia. A 5- or 6-French guide catheter was introduced in the femoral artery and placed in the internal carotid or vertebral artery.

Angiography was then performed, followed by superselective catheterization of the AVM feeders, using an Ultraflow HPC™ microcateter (1.5 Fr) (*Micro Therapeutics, Inc*., Irvine, CA, USA), which allowed a better understanding of the AVM angioarchitectural findings. Intranidal catheter position was achieved whenever it was feasible. Lipiodol and Histoacryl (*Braun Melsugen SA*, S. Gonçalo, RJ, Brazil) were used as agents of embolization. No more than one-third of the lesion was embolized per session. Every effort was made to target feeders harboring intranidal aneurysms. In cases that need additional sessions, the interval between them was at least 30 days. Dynamic subtraction fluoroscopy was employed to display the nidus and feeders, as well as the cast.

### Radiosurgery

The planning was done using *Brain Scan, Image Fusion 5.3.1* (*BrainLab*, Munich, Germany) based on the combination of the angiographic data, contrast-enhanced CT images, and thin-sliced MRI. The selection of the radiation dose dependent on location and target volume. The maximal dose varied from 1200 to 2500 cGy, at an isodose of 80%. No difficulties related to the previous partial embolization of the lesion were observed. Radiation was delivered using Varian 6 MV system (*Varian, Inc*., Palo Alto, CA, USA).

### Follow-up

Patients were followed up with clinical examinations for at least 3 years after treatment. Clinical follow-up was carried out in the 3rd, 6th, and 12th months, and once per year thereafter. MRI was used to follow the patients once per year. Cerebral angiography was performed 3 years after treatment.

### Statistical Analysis

Mann–Whitney test, “Student’s *t*-test,” and χ^2^ tests were used for statistical analysis, as appropriate. The level of significance was determined at *p* < 0.05.

## Results

Arteriovenous malformations were located in the frontal lobe (*n* = 18; 20%), parietal lobe (*n* = 15; 16.7%), basal ganglia (*n* = 15; 16.7%), thalamus (*n* = 12; 13.3%), temporal lobe (*n* = 7; 7.8%), ventricles (*n* = 7; 7.8%), occipital lobe (*n* = 6; 6.7%), insula (*n* = 5; 5.6%), cerebellum (*n* = 4; 4.4%), and corpus callosum (*n* = 1; 1.1%).

Table 1 summarizes the clinical data. Motor deficit was presented in 41 patients (45.6%), whereas asymptomatic cases were composed of 7.8% (*n* = 7). Forty-nine patients (60%) presented grade III AVMs. Embolization significantly reduced the AVM diameter and AVM’s grade. Embolized AVMs presented greater volume than non-embolized ones (*p* = 0.013). Irradiation dose was significantly smaller in the embolized group (*p* = 0.029) (Table 3).

**Table 3:** **Volume before RDS and irradiation does in the two groups**.

	Embolization	*n*	Mean	SD	Values		*p*
					Minimum	Maximum	
Volume before RDS	No	31	8.17	8.66	0.33	33.60	0.013^a^
	Yes	59	13.36	10.63	0.38	52.54	
Irradiation dose	No	31	1726.00	314.87	1300.00	2500.00	0.029^b^
	Yes	59	1686.00	266.56	1200.00	2500.00	

*^a^Mann–Whitney test*.

*^b^Student’s t-test*.

*RDS, radiosurgery*.

Permanent complications occurred in eight (13.5%) patients in the embolization group (Table 4). Two motor deficits were related to bleeding. Ischemic events occurred in six patients. Transient clinical complications occurred in seven patients (11.7%) in the embolized group (Table 5). Complications in the non-embolized group were related with biological effects of radiation and were managed using corticoids. Delayed radiation induced a permanent complication in one patient. Two patients experienced hemorrhage during the latency period without related neurological deficits. Additional radiosurgical procedure was performed in 7 (22.6%) in the non-embolized group and in 25 patients (42.4%) of the embolized group.

**Table 4:** **Permanent clinical complications in the embolized group**.

Clinical complications	*n*	%
Hemiparesis	4	6.7
Hemiparesis + disfasia	1	1.6
Hemifacial anesthesia + diplopia + vertigo	1	1.6
Hemiparesis + disartria + cerebellar syndrome	1	1.6
Deafness	1	1.6

**Table 5:** **Transient clinical complications in the embolized group**.

Clinical complications	*n*	%
Hemiparesis	5	8.4
Seizures	1	1.6
Wallenberg syndrome	1	1.6

No significant differences in final morbidity, postprocedural radiological findings, rate of occlusion, and need for additional procedures were observed between the two groups.

## Discussion

Grades I and II AVMs may be effectively treated either by surgery or radiosurgery; however, management of larger lesions represents a formidable challenge and most often requires multiple treatment strategies (5, 14, 20, 23, 24). In this setting, initial endovascular embolization, aiming to decrease AVM’s size, combined with stereotactic radiosurgery represents an empirical option. Nonetheless, the benefits and advantages of preradiosurgical embolization have not been defined thus far (2, 3, 9, 11, 24). Indeed, the drawbacks of preradiosurgical embolization have been often emphasized (2, 3, 9, 11, 20).

In several studies, preradiosurgical nidus embolization was associated with lower obliteration rate, higher morbidity, and worse outcomes (18). Andrade-Souza et al. (25) demonstrated that obliteration occurred in 47% of patients treated with embolization compared with 70% of the radiosurgery-alone group. Schlienger et al. (18) verified that the obliteration rate reaches 54% in cases with preradiosurgical embolization and 71% in radiosurgery-alone cohort. Other study showed obliteration rates of 26 and 76% for embolized and non-embolized lesions, respectively (15). Pollock et al. found that preradiosurgical embolization was considered as a negative factor for obliteration and optimal results (26). Xu et al. meta-analyzed 10 studies that demonstrated no benefits for preradiosurgical embolization; however, there were significant publication bias (27).

Various factors have been considered as responsible for such unsatisfactory outcomes. Indeed, ineffective embolization without reduction of AVM’s total volume may difficult radiosurgical targeting (6, 11, 22). Moreover, partial embolization may cause dissociation of the AVM into distinct compartments, impairing conformal radiosurgical planning (6, 11, 18, 21).

Conversely, other studies have shown different conclusions. Izawa et al. (11) reported that preradiosurgical embolization did not result in improved occlusion or higher complications rates. Indeed, it may facilitate complete coverage with effective dose. Blackburn et al. (2) conclude that staged preradiosurgical endovascular embolization provides an effective means of treating large AVMs not amenable to surgical or radiosurgical-alone treatments. The outcomes and complication rates compare favorably to the results of other reported therapeutic strategies (2).

**Figure 1:**
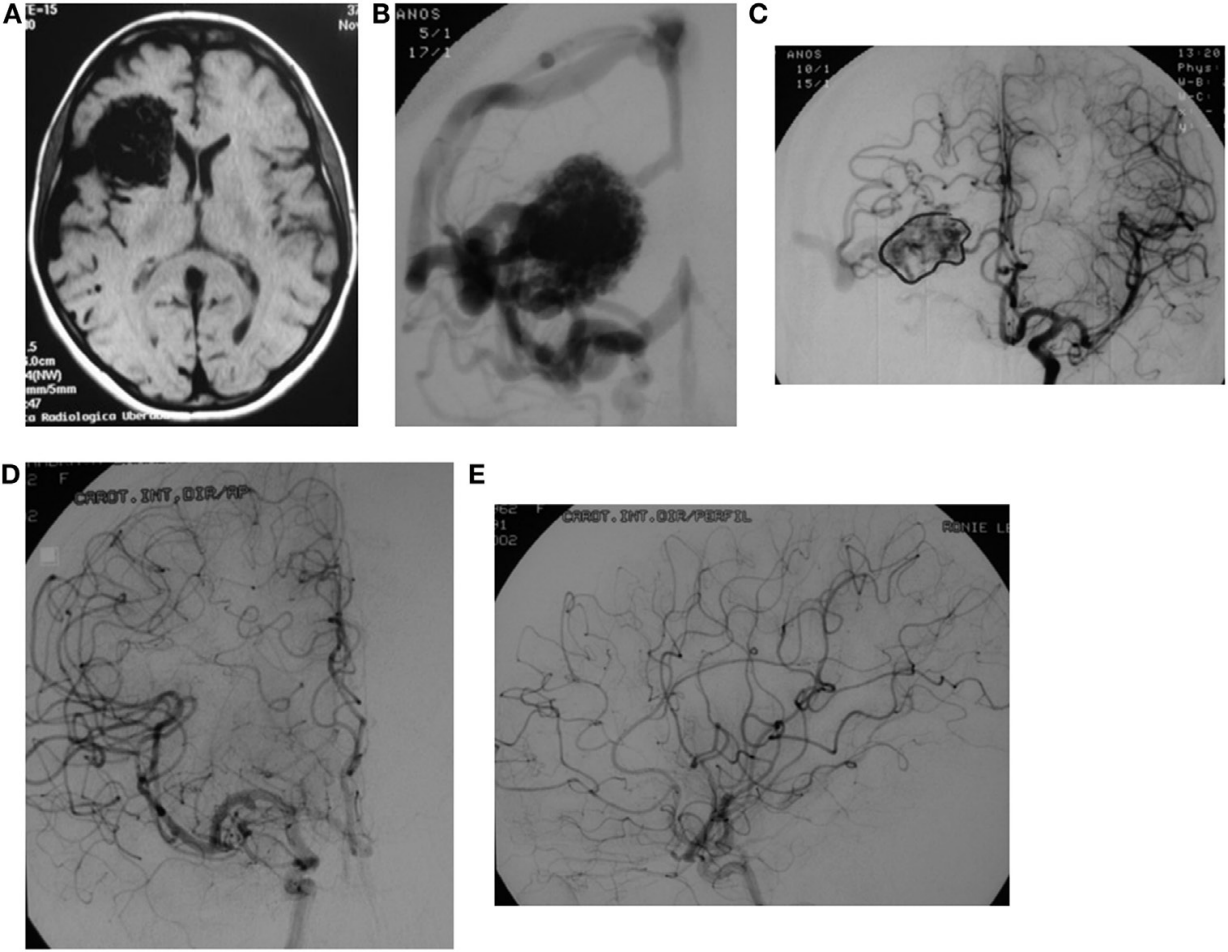
**A 37-year-old female presents with a grade IV frontal lobe AVM**. **(A)** RMI depicts a right frontal lobe AVM. **(B)** Anteroposterior angiogram view. **(C)** Anteroposterior angiogram view after embolization displays a significant reduction of the AVM’s size. Anteroposterior **(D)** and lateral **(E)** angiogram views conveying total obliteration of the AVM 2 years radiosurgery (1700 cGy).

**Figure 2:**
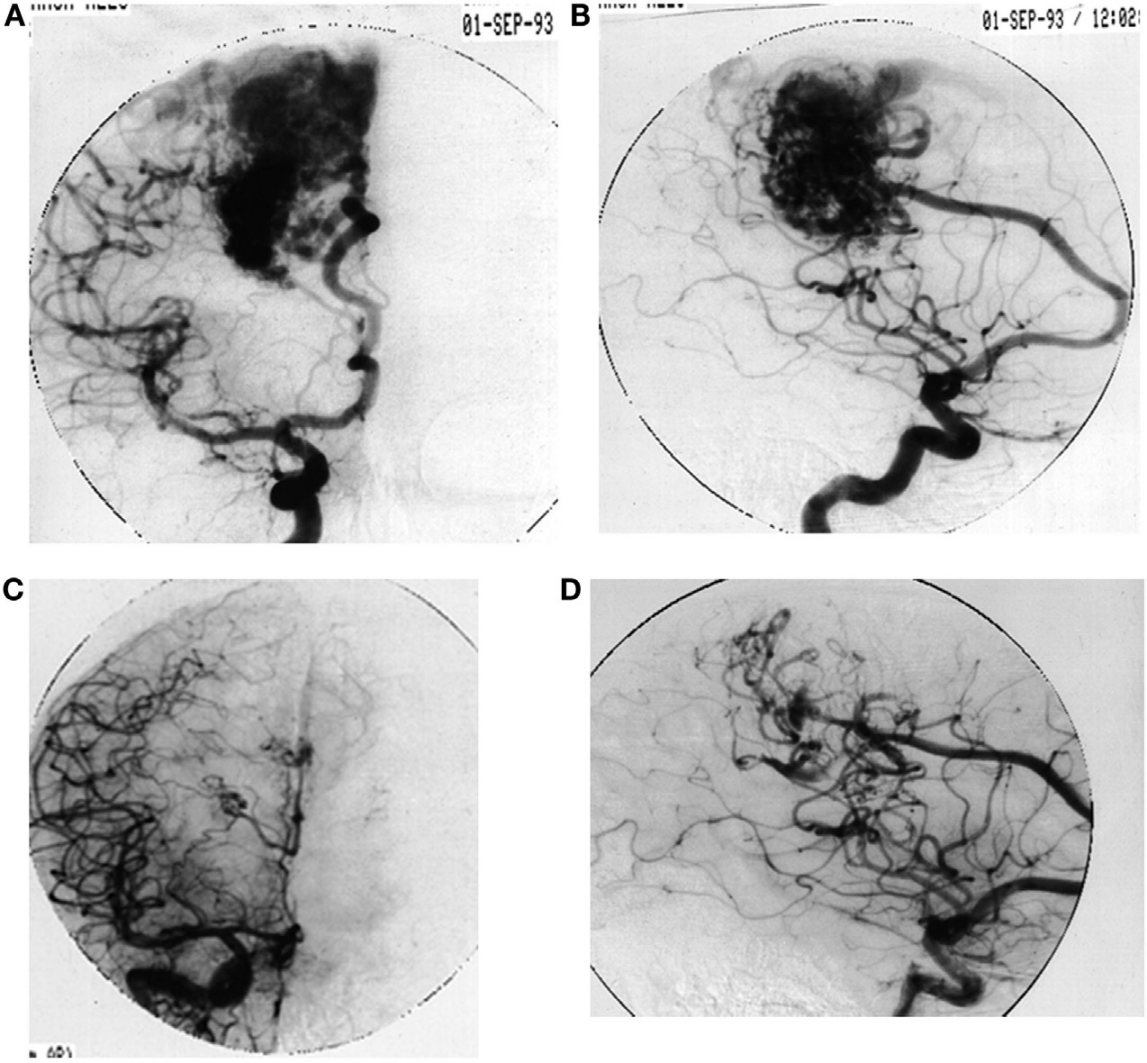
**A 21-year-old male with a parietal lobe hemorrhage**. Angiogram diagnosed a grade IV AVM. **(A)** Anteroposterior view. **(B)** Lateral view. Four years after radiosurgery (2200 cGy) angiogram evidenced total occlusion. **(C)** Anteroposterior view. **(D)** Lateral view.

In this study, the obliteration rate and long-term morbidity after combined management of AVMs with embolization and radiosurgery do not significantly differ. The greater mean nidus volume in the embolization group may imply that embolization was mainly indicated in larger lesions, and therefore, it can be speculated that reduction of the nidus’ size might play an important role in the overall effectiveness of treatment. Large AVMs were converted to smaller ones, feasible to radiosurgery and otherwise untreatable lesions (Figures 1 and 2).

Notwithstanding, combined management may result in associating the intrinsic morbidity of both procedures (11). Nonetheless, no major neurologic deterioration was observed after embolization, probably reflecting careful case selection and non-aggressive strategy of endovascular management. Aggressive embolization of a large AVM in a single session may augment the risks (5, 11, 22, 23, 25, 28).

The reported rate of long-term postradiosurgical complications in previously embolized cases was higher, equal (9, 11), or lower (2, 13) than in cases treated with radiosurgery alone (11). In our study, the long-term morbidity rate did not differ significantly between the groups with and without previous embolization.

A substantial benefit of preradiosurgical embolization was the overall reduction of the irradiation dose, even considering the additional radiation dose required in further radiosurgical procedures. Significant reduction of size was probably responsible for the complete coverage of the nidus with the effective dose. Even though, the occlusion rates did not differ significantly, several lesions that could be left untreated were effectively managed and occluded.

### Limitations of the Study

This is a retrospective study, and as such, it is susceptible to systematic errors (bias). In the embolized group, AVMs were larger than in radiosurgery-only group, which may introduce selection bias, even though, there were no statistical differences between the two groups. A subgroup analysis has not been performed, for small or large AVMs, due the insufficient number of cases, which may underpower the study.

In this series, mean patient age was 30.6 years. The follow-up period of 3 years is relatively short and may be difficult to make definitive recommendations for this particular group, as younger patients have higher chance of rebleed in the long term, after non-surgical treatment.

We found no statistical differences regarding percentage of occlusion and need for further procedures, results that are different from other studies and which may represent an additional contribution to the literature in this controversial matter.

## Conclusion

This study has demonstrated that partial embolization of intracranial AVMs before stereotactic radiosurgery does not result in statistically significant impaired obliteration rate compared with cases treated with radiosurgery alone. Preradiosurgical embolization may present benefits of reducing size of the AVMs and may facilitate the coverage of the AVM with the effective irradiation dose. Thus, combined management may be effective for selected and otherwise deemed untreatable cases.

## Ehtics Statement

The study was approved by the Comissão de Etica para Aanalise de Projetos de Pesquisa (CAPPesq) – 612/06. All patients signed an informed consent form.

## Author Contributions

All the authors participated in the revision and supervision of the manuscript.

## Conflict of Interest Statement

The authors declare that the research was conducted in the absence of any commercial or financial relationships that could be construed as a potential conflict of interest.
